# Association Between Prophylactic Anti‐Seizure Medication and Early Post‐Traumatic Seizures: An International Observational Multicenter Study

**DOI:** 10.1002/mco2.70524

**Published:** 2025-12-10

**Authors:** Jian Ji, Juan D. Roa G, Shu‐Ling Chong, Quan Wang, Chin Seng Gan, Jane P. W. Ng, Thelma Elvira Teran Miranda, Freddy Israel Pantoja Chamorro, Lawrence Chi Ngong Chan, Qalab Abbas, Jacqueline S. M. Ong, Ivan J. Ardila, Yasser M. Kazzaz, Jesús A. Domínguez‐Rojas, Hiroshi Kurosawa, Susana Beatriz Reyes Domínguez, Natalia Elizabeth Gómez Arriola, Natalia Zita Watzlawik, Adriana Yock‐Corrales, Rubén Eduardo Lasso Palomino, Gabriela Aparicio, Paula Caporal, Rosa Elena de la Torre Gómez, Chunfeng Liu, Rujipat Samransamruajkit, Nattachai Anantasit, Deborah M. Turina, Marisol Fonseca Flores, Pei‐Chuen Lee, Francisco J. Pilar‐Orive, Hongxing Dang, Yek Kee Chor, Meixiu Ming, Juan C. Jaramillo‐Bustamante, Sebastián González‐Dambrauskas, Jan Hau Lee, Suyun Qian

**Affiliations:** ^1^ Pediatric Intensive Care Unit Beijing Children's Hospital Capital Medical University National Center for Children's Health Beijing China; ^2^ Pediatric Collaborative Latin American Network (LARed Network) Pediatric Intensive Care Unit Los Cobos Medical Center Universidad Del Bosque Bogotá Colombia; ^3^ Department of Emergency Medicine KK Women's and Children's Hospital Singapore SingHealth Duke‐NUS Global Health Institute, Duke‐NUS Medical School Singapore Singapore; ^4^ Department of Pediatrics University Malaya Medical Centre Kuala Lumpur Malaysia; ^5^ KK Research Centre KK Women's and Children's Hospital Singapore Singapore; ^6^ Department of Pediatrics Hospital Del Niño Manuel Ascencio Villarroel Cochabamba Bolivia; ^7^ Intensive Care Unit Hospital Infantil Los Angeles Pasto Colombia; ^8^ Department of Pediatrics Prince of Wales Hospital The Chinese University of Hong Kong Hong Kong SAR China; ^9^ Department of Pediatrics and Child Health Aga Khan University Hospital Karachi Pakistan; ^10^ Department of Pediatrics Yong Loo Lin School of Medicine Khoo Teck Puat National University Children's Medical Institute National University Hospital Singapore National University of Singapore Singapore Singapore; ^11^ Pediatric Critical Care Clinica UROS Neiva Colombia; ^12^ Department of Pediatrics King Abdullah International Medical Research Center Riyadh Saudi Arabia; ^13^ Department of Pediatrics and Pediatric Critical Care Medicine Hospital Nacional Hipolito Unanue El Agustino Peru Pediatric Chapter of the Peruvian Society of Intensive Care Medicine Lima Peru; ^14^ Department of Pediatric Critical Care Medicine Hyogo Prefectural Kobe Children's Hospital Kobe Japan; ^15^ Pediatric Intensive Care Unit Pediatric Department Virgen De La Arrixaca Hospital Murcia Spain; ^16^ Emergency Department Hospital Del Trauma Asunción Paraguay; ^17^ Department of Pediatric Intensive Care Unit Hospital De Pediatric Garrahan Ciudad Autónoma de Buenos Aires Buenos Aires Argentina; ^18^ Emergency Department National Children's Hospital “Dr. Carlos Saenz Herrera,” CCSS San José Costa Rica; ^19^ Pediatric Intensive Care Unit Fundación Valle del Lili Cali Valle del Cauca Colombia; ^20^ Pediatric Critical Care Unit Hospital De Ninos de La Plata Sor María Ludovica Buenos Aires Argentina; ^21^ Pediatric Intensive Care Unit Children's Hospital “Sor Maria Ludovica Buenos Aires Argentina Pediatric Collaborative Latin American Network (LARed Network) Buenos Aires Argentina; ^22^ Hospital Civil de Guadalajara Guadalajara Mexico; ^23^ Pediatric Department Shengjing Hospital China Medical University Shenyang Liaoning China; ^24^ Department of Pediatrics Pediatric Critical Care Division King Chulalongkorn Memorial Hospital Chulalongkorn University Bangkok Thailand; ^25^ Pediatric Critical Care Division Department of Pediatrics Ramathibodi Hospital Bangkok Thailand; ^26^ Pediatric Intensive Care Unit Ricardo Guiterrez Children's Hospital Buenos Aires Argentina; ^27^ Pediatric Critical Care Mexican Institute of Social Security México City Mexico; ^28^ Pediatrics Department UKM Specialist Children's Hospital, Kuala Lumpur Wilayah Persekutuan Malaysia; ^29^ Department PICU Hospital Universitario de Cruces Barakaldo Spain; ^30^ Department of Pediatric Intensive Care Unit Children's Hospital of Chongqing Medical University Chongqing China; ^31^ Department of Pediatrics Sarawak General Hospital Sarawak Malaysia; ^32^ Department of Pediatric Intensive Care Unit Children's Hospital of Fudan University Shanghai China; ^33^ Pediatric Intensive Care Unit General Hospital of Medellín “Luz Castro De Gutiérrez Medellín Colombia; ^34^ Pediatric Collaborative Latin American Network (LARed Network) Department of Pediatrics and Pediatric Intensive Care Unit School of Medicine Hospital Pereira Rossell University of the Republic Montevideo Uruguay; ^35^ Children's Intensive Care Unit KK Women's and Children's Hospital Singapore Singapore

## Abstract

Evidence for the use of prophylactic anti‐seizure medication (ASM) in traumatic brain injury (TBI) in reducing the occurrence of early post‐traumatic seizure (EPTS) remains equivocal. This study aimed to analyze the prevalence of EPTS in children with TBI, compare clinical characteristics of those with and without EPTS, and explore the association between prophylactic ASM and EPTS. We performed an observational study among 28 pediatric intensive care units in 15 countries from January 2014 to October 2022. The rate of EPTS was compared between individuals prescribed prophylactic ASM and those who were not. Logistic regression was used to examine the association between ASM and EPTS. Among 697 children with TBI, 161 (23.1%) developed EPTS and 280 (40.2%) received prophylactic ASM treatment. Use of prophylactic ASM was associated with a lower likelihood of developing EPTS (27/280 (9.6%) vs. 134/417 (32.1%), *p* < 0.001). The most frequently used prophylactic ASMs were phenytoin, levetiracetam, and phenobarbital. Age ≤ 4 years and GCS ≤ 8 were associated with increased odds of developing EPTS (aOR 2.29, 95% CI 1.54–3.40, *p* < 0.001 and aOR 1.80, 95% CI 1.18–2.74, *p =* 0.01). Our data provide evidence supporting the potential protective role of prophylactic ASM against EPTS.

## Graphical abstract

**Multicenter study**: Data from 28 Pediatric Intensive Care Units (PICUs) across 15 countries in Asia, Latin America, and Europe.

**Key findings**:

– Incidence of EPTS: 23.1% of children with TBI developed early post‐traumatic seizures (EPTS).

– Effectiveness of Prophylactic ASM: 9.6% of children who received prophylactic anti‐seizure medication (ASM). developed EPTS, compared to 32.1% in those without ASM.

– Regional Differences: There is variation in ASM usage and EPTS rates between regions (Asia, Latin America, and Europe).

**Conclusion**: The use of prophylactic ASM significantly reduced the odds of developing EPTS in children with traumatic brain injury, with varying medication preferences across regions.

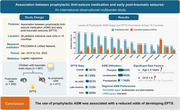

## 1 Introduction

Traumatic brain injury (TBI) is defined as structural or functional brain damage resulting from external mechanical forces, such as blunt trauma, penetrating injuries, rapid acceleration‐deceleration (e.g., motor vehicle accidents), or blast waves, which leads to disruption of normal brain function [1, 2]. Globally, TBI ranks among the most prevalent neurological conditions, with an estimated 50–60 million new cases annually and an economic burden of approximately $400 billion, placing it within the top three causes of injury‐related death and disability projected to persist until at least 2030 [3].

Children face heightened vulnerability to TBI, which represents a major cause of pediatric hospitalization [4]. Moreover, children have a significantly increased risk of psychiatric issues after TBI compared with those after an orthopedic injury [5]. Early post‐traumatic seizures (EPTS)—occurring within 7 days of injury—constitute a significant complication [6]. Data showed that the rate of EPTS in children with moderate‐to‐severe TBI is about 24.3%–34% [7, 8]. This clinical burden is compounded by inconsistent TBI care practices across acute and post‐acute phases worldwide, with substantial variability documented among centers, regions, and countries [9].

Anti‐seizure medication (ASM) is generally prescribed to treat EPTS for a short period of time; however, the role of prophylactic ASM remains equivocal. The 2019 publication of the latest iteration of guidelines reassessed the evidence supporting the use of prophylactic phenytoin and levetiracetam in preventing EPTS. Recent Class III studies have influenced a Level III recommendation endorsing prophylactic treatment for mitigating EPTS occurrence [10]. The varying degrees of uncertainty regarding efficacy result in diverse clinical practices concerning the prophylactic use of ASM in pediatric TBI [11]. Additionally, the current evidence is primarily derived from data originating in North America and Europe [10]. The incidence and risk factors of EPTS in trauma centers and pediatric intensive care units (PICUs) in Asia and Latin America are not extensively documented. Hence, there is an urgent requirement for further extensive clinical data collection to strengthen the evidence for addressing this matter. The first Lancet Neurology Commission on TBI, published in 2017, emphasized that such inter‐center heterogeneity in TBI management and outcomes creates unique opportunities for comparative‐effectiveness research to strengthen evidence‐based protocols [3]. Nevertheless, current diagnostic and patient classification methods remain insufficient for precision targeting therapies to individual needs.

Using data from a collaborative multi‐center study across Asia, Latin America, and Europe, we aimed to analyze the prevalence of EPTS in children with TBI, compare clinical characteristics between children who developed EPTS and those who did not, and explore the association between use of prophylactic ASM and subsequent development of EPTS. We aim to provide clinically implementable evidence to address global disparities in EPTS prevention and inform evidence‐based therapeutic optimization for vulnerable pediatric populations.

## 2 Results

### 2.1 Clinical Characteristics of the TBI Children

**FIGURE 1:**
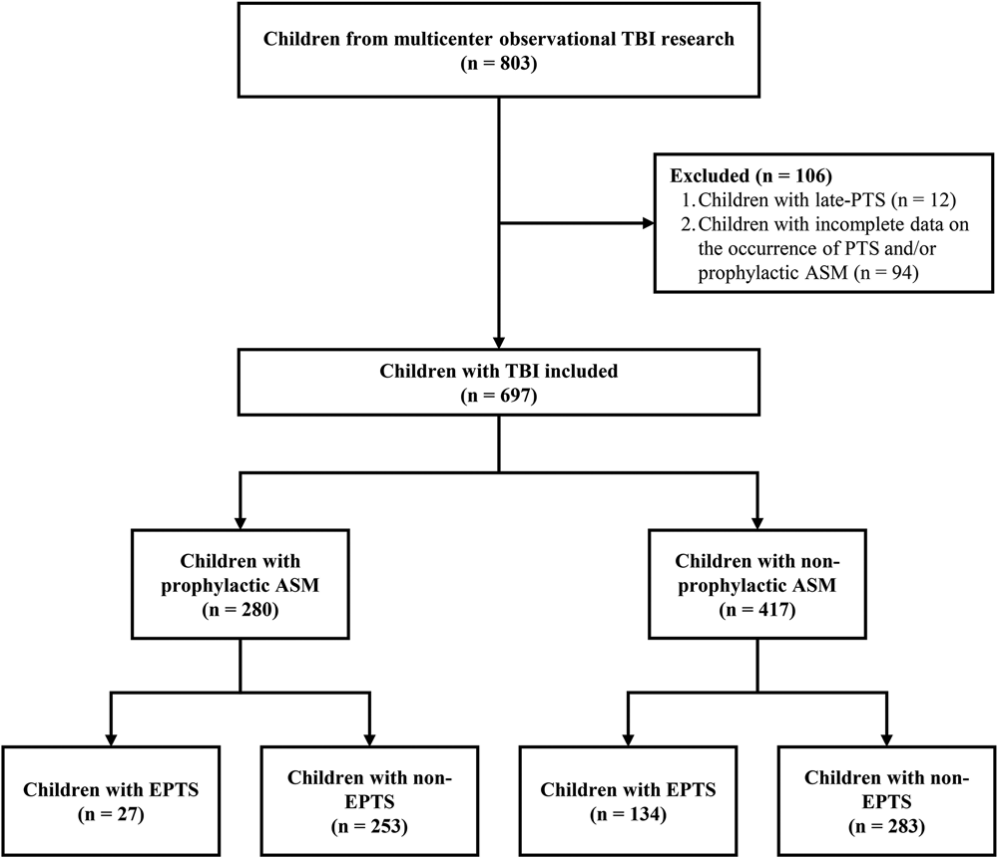
Flowchart of participant selection and stratification in the multicenter observational study on TBI in children. From an initial cohort of 803 children, 106 were excluded due to late PTS (*n* = 12) or incomplete data on PTS occurrence and/or ASM (*n* = 94). The remaining 697 children with TBI were stratified by prophylactic ASM use: 280 received prophylactic ASM (of whom 27 developed EPTS and 253 did not), while 417 did not receive prophylactic ASM (of whom 134 developed EPTS and 283 did not). TBI, traumatic brain injury; PTS, post‐traumatic seizures; ASM, anti‐seizure medication; EPTS, early post‐traumatic seizures.

Among a total of 803 children from 28 PICUs, 94 (94/803, 11.7%) children were excluded due to incomplete data and 12 (12/803, 1.5%) children due to late PTS (Figure 1). We analyzed 697 pediatric TBI cases with an average age of 5.8 years (SD 4.9) and a GCS reported as a median of 8 (IQR: 5–11). Detailed information is presented in Table 1. In summary, EEG monitoring was used in 17 PICUs (60.7%) involving 208 patients. These patients had a mean GCS score of 7.0 (vs. 8.5 in the non‐monitored group) and a mean age of 4.5 years (vs. 6.3 years), with no statistically significant differences. The monitored group included 193 Asian patients and 15 South American patients.

**TABLE 1:** Baseline information of the patients (total N = 697).

Characteristics	Cases (*N*)	Percentage (%)
Age		
≤4 years	355	50.9% (697)
Sex		
Male	458	65.7% (697)
GCS at hospital admission		
Mild (GCS 13–15)	117	16.8% (697)
Moderate (GCS 9–12)	161	23.1% (697)
Severe (GCS ≤ 8)	419	60.1% (697)
Mechanism of injury		
Road traffic collision	309	44.3% (697)
Fall	282	40.4% (697)
Abusive head trauma	49	7.2% (697)
Others	57	8.1% (697)
EPTS	161	23.1% (697)
Initial day after TBI	100	62.1% (161)
Day 2 after TBI	21	13.0% (161)
Day 3 after TBI	11	6.8% (161)
Day 4 after TBI	7	4.4% (161)
Day 5 after TBI	9	5.6% (161)
Day 6 after TBI	6	3.7% (161)
Day 7 after TBI	7	4.4% (161)
Mortality in hospital	80	11.5% (161)
Mortality within 7 days of hospital admission	58	72.5% (80)
Multiple trauma	331	47.5% (697)
Skull fracture	155	22.2% (697)
Intracranial hemorrhage	469	67.3% (697)
Penetrating TBI	19	2.7% (697)
Clinical treatment		
Hyperosmolar therapy	487	69.9% (697)
Hyperventilation	64	9.2% (697)
Neurosurgical intervention	291	41.8% (697)
Prophylactic ASM	280	40.2% (697)
Combined prophylactic therapy	8	2.9% (697)
Monotherapy	272	97.1% (697)
EEG	208	29.8% (697)

Abbreviations: ASM, anti‐seizure medication; EEG, electroencephalogram; GSC, Glasgow Coma Scale; PTS, post‐traumatic seizures; TBI, traumatic brain injury.

### 2.2 Comparison of the TBI Children With EPTS and Without EPTS

In the study, 161 children were identified with EPTS, representing 23.1% of the total sample. Among them, 155 presented with clinical seizures, while six displayed subclinical seizures. The incidence of EPTS was 25.6% (125/488) in the Asia region, compared to 17.8% (34/191) in Latin America, demonstrating a statistically significant discrepancy (*p* = 0.031). Detailed clinical information is available in Table 2. A significantly higher proportion of children aged ≤ 4 years was observed among those who developed EPTS compared with those who did not (110/161 [68.3%] vs. 245/536 [45.7%]; *p* < 0.001).

**TABLE 2:** Comparison of information between EPTS and non‐EPTS groups.

Variables	EPTS (*N* = 161)	Non‐EPTS (*N* = 536)	*p*‐value
Sex			0.881
Male	105 (65.2%)	353 (65.9%)	
Age (years)			<0.001
≤4years	110 (68.3%)	245 (45.7%)	
TBI severity at hospital admission			0.068
Mild (GCS 13–15)	18 (15.4%)	99 (84.6%)	
Moderate (GCS 9–12)	36 (22.4%)	125 (77.6%)	
Severe (GCS ≤ 8)	107 (25.5%)	312 (74.5%)	
Multiple trauma	69 (20.8%)	262 (79.2%)	0.180
Surgical intervention	70 (24.1%)	221 (75.9%)	0.612
Hyperventilation	11 (17.2%)	53 (82.8%)	0.239
Mechanism of injury			<0.001
Road traffic collision	52 (16.8%)	257 (83.2%)	
Fall	65 (23.0%)	217 (77.0%)	
Abusive head trauma	30 (61.2%)	19 (38.8%)	
Others	14 (24.6%)	43 (75.4%)	
Skull fracture	28 (18.1%)	127 (81.9%)	0.092
Intracranial hemorrhage	100 (21.3%)	369 (78.7%)	0.110
Penetrating TBI	2 (10.5%)	17 (89.5%)	0.274
Target temperature management	110 (21.8%)	394 (78.2%)	0.197
Hypertonic therapy	106 (21.8%)	381 (78.2%)	0.200
Prophylactic ASM	27 (9.6%)	253 (90.4%)	<0.001

Abbreviations: ASM, anti‐seizure medication; GSC, Glasgow Coma Scale; TBI, traumatic brain injury.

### 2.3 Association Between Prophylactic ASM and Subsequent EPTS in Children With TBI

Of 697 children, 280 (40.2%) received prophylactic ASM. A lower proportion of children who received prophylactic ASM (27/280, 9.6%) developed EPTS, compared to 134/417 (32.1%) children in the group without prophylactic ASM (*p* < 0.001). In the prophylaxis group, children exhibited notably elevated incidences of GCS ≤ 8 (median 8 [range 4–8]), skull fractures, utilization of hypertonic therapy, and requirement for neurosurgical interventions in comparison to the non‐prophylaxis group (median 8 [range 6–10]), as illustrated in Table 3.

**TABLE 3:** Comparison of information between prophylaxis and non‐prophylaxis groups.

Characteristics	Prophylaxis group (*N* = 280)	Non‐prophylaxis group (*N* = 417)	*p*‐value
Sex			0.035
Male	171 (61.1%)	287 (68.8%)	
Age			
≤ 4 years	129 (46.1%)	226 (54.2%)	0.035
TBI severity at hospital admission			0.194
Mild (GCS 13–15)	39 (33.3%)	78 (66.7%)	
Moderate (GCS 9–12)	63 (39.1%)	98 (60.9%)	
Severe (GCS ≤ 8)	178 (42.5%)	241 (57.5%)	
Mortality in hospital	25 (8.9%)	55 (13.2%)	0.084
Multiple trauma	162 (57.9%)	213 (51.1%)	0.078
Skull fracture	67 (23.9%)	88 (21.1%)	<0.001
Intracranial hemorrhage	201 (42.9%)	268 (57.1%)	0.038
Penetrating TBI	13 (68.4%)	6 (31.6%)	0.011
Mechanism of injury			<0.001
Road traffic collision	134 (43.4%)	175 (56.6%)	
Fall	114 (40.4%)	168 (59.6%)	
Abusive head trauma	7 (14.3%)	42 (85.7%)	
Others	25 (43.9%)	32 (56.1%)	
Clinical treatment			
Hypertonic therapy	213 (76.1%)	274 (65.7%)	0.003
Hyperventilation	27 (9.6%)	37 (8.9%)	0.730
Neurosurgical intervention	136 (48.6%)	155 (37.2%)	0.003
EEG	57 (20.4%)	151 (36.2%)	0.285
EPTS	27 (9.6%)	134 (32.1%)	<0.001

Abbreviations: ASM, anti‐seizure medication; EEG, electroencephalogram; EPTS, early post‐traumatic seizures; GSC, Glasgow Coma Scale; TBI, traumatic brain injury.

In the multivariable analysis, the use of prophylactic ASM therapy was associated with reduced odds of developing EPTS (aOR 0.21, 95% CI 0.13–0.34, *p* < 0.001). Age ≤ 4 years and GCS ≤ 8 were associated with increased odds of developing EPTS (aOR 2.29, 95% CI 1.54–3.40, *p* < 0.001 and aOR 1.80, 95% CI 1.18–2.74, *p =* 0.01) (Table 4).

**TABLE 4:** Multivariable logistic regression analysis of risk factors for EPTS.

Characteristics	Unadjusted OR (95% CI)	Unadjusted *p* value	Adjusted OR (95% CI)	Adjusted *p* value	VIF
≤ 4 years old	2.56 (1.76, 3.72)	<0.001	2.29 (1.54, 3.40)	<0.001	1.075
GCS ≤ 8	1.42 (0.98, 2.06)	0.060	1.80 (1.18, 2.74)	0.010	1.138
Skull fracture	0.68 (0.43, 1.07)	0.090	0.81 (0.50, 1.32)	0.400	1.062
Intracranial hemorrhage	1.39 (0.94, 2.05)	0.100	1.39 (0.91, 2.11)	0.130	1.021
Multiple trauma	0.78 (0.55, 1.12)	0.180	0.71 (0.47, 1.08)	0.110	1.201
Hypertonic therapy	0.78 (0.54, 1.14)	0.200	0.94 (0.61, 1.44)	0.770	1.164
Temperature control	0.78 (0.53, 1.14)	0.200	0.99 (0.64, 1.51)	0.940	1.096
Prophylactic ASM	0.23 (0.14, 0.35)	<0.001	0.21 (0.13, 0.34)	<0.001	1.098

Abbreviations: ASM, anti‐seizure medication; GSC, Glasgow Coma Scale; VIF, variance inflation factor.

Sensitivity analyses confirmed the robustness of these findings. Results remained consistent in analyses stratified by geographic region and in the subgroup restricted to EEG‐monitored patients. In the PACCMAN region (*n* = 488), prophylactic ASM was protective (aOR 0.46, 95% CI 0.39–0.53, *p* < 0.001), with age ≤ 4 years (aOR 2.96, 95% CI 1.59–5.65, *p* < 0.001) and GCS ≤ 8 (aOR 1.53, 95% CI 1.31–1.75, *p* < 0.001) as risk factors (Table S1). Similarly, in the LARed cohort (*n* = 209), prophylactic ASM demonstrated a protective effect (aOR 0.26, 95% CI 0.19–0.35, *p* < 0.001), while age ≤ 4 years (aOR 2.23, 95% CI 1.10–3.65, *p* < 0.001) and GCS ≤ 8 (aOR 2.33, 95% CI 1.89–2.75, *p* < 0.001) were associated with increased risk (Table S2). In the EEG‐confirmed cases (*n* = 208), prophylactic ASM remained protective (aOR 0.23, 95% CI 0.16–0.30, *p* < 0.001), while age ≤ 4 years (aOR 2.24, 95% CI 1.94–2.48, *p* = 0.021) and GCS ≤ 8 (aOR 1.80, 95% CI 1.18–2.74, *p* = 0.010) were associated with increased risk (Table S3).

### 2.4 Selection of Prophylactic ASMs and Their Effectiveness in EPTS Prevention

Among 272 children who received monotherapy, phenytoin was the most widely used (124/272, 45.6%), followed by levetiracetam (109/272, 40.0%) and phenobarbital (39/272, 14.4%). A subgroup analysis conducted on 272 patients in the prophylactic anti‐seizure monotherapy group showed that five of 124 (4.0%) patients who received phenytoin, nine of 109 (8.3%) patients who received levetiracetam, and 10 of 39 (25.6%) patients who received phenobarbital experienced EPTS (*p* < 0.001). After adjusting for relevant clinical variables in a multifactorial analysis, significant differences were observed among the three medications. Specifically, levetiracetam and phenytoin exhibited heightened protective efficacy compared to phenobarbital, as detailed in Table S4.

### 2.5 Regional Differences in the Use of ASM

**FIGURE 2:**
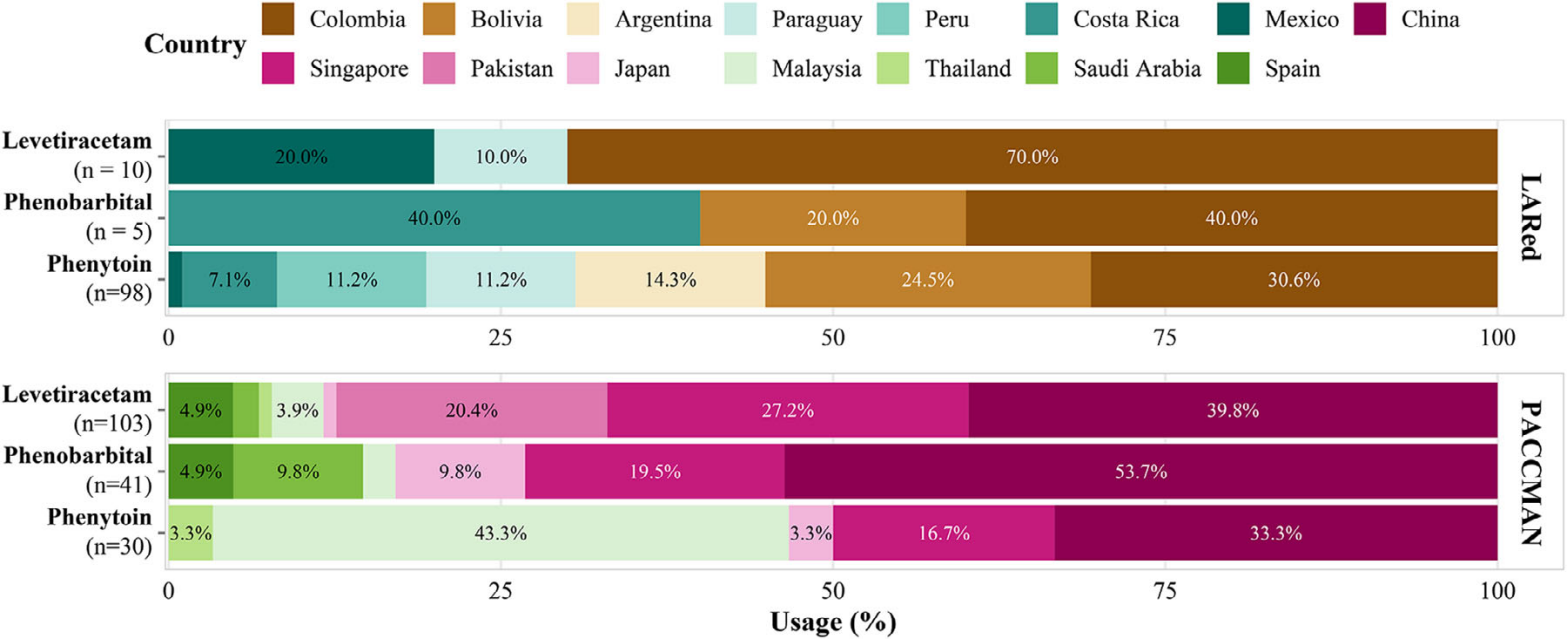
Distribution of primary preventive antiepileptic drug usage by country in PACCMAN and LARed. Each facet corresponds to one of the regions (PACCMAN and LARed), with the vertical axis representing the three antiepileptic drugs (phenytoin, phenobarbital, and levetiracetam) and the horizontal axis showing the percentage distribution. Each bar illustrates the case composition for a specific drug within the region, stacked by country, where the total composition for each region‐drug pair sums to 100%. The bars are color‐coded by country, with only values exceeding 3% labeled within each bar. PACCMAN, Pediatric Acute and Critical Care Medicine Asian Network; LARed, Red Colaborativa Pediátrica de Latinoamérica.

The study included 697 participants, 488 of whom were from Asia. Among them, 161 (33.0%) received prophylactic ASM. Additionally, there were 191 participants from Latin America, with 112 (58.6%) undergoing prophylactic antiepileptic therapy. A statistically significant difference was noted between the Asian and Latin American groups (*p* < 0.001). There was a preference for levetiracetam and phenobarbital as primary prophylactic ASM in countries within the PACCMAN region (notably China, Singapore and Pakistan). China reported the highest proportion (33.7%) of cases, with levetiracetam and phenobarbital being used almost equally. Following China (41/103, 39.8%), Singapore (28/103, 27.2%) and Pakistan (21/103, 20.4%) ranked second and third, respectively, in levetiracetam usage. Phenobarbital emerged as the second most used ASM in Asia, with China (22/41, 53.7%), Singapore (8/41, 19.5%), Saudi Arabia (4/41, 9.8%), and Japan (4/41, 9.8%) demonstrating the highest utilization rates. In contrast, study sites in the LARed group, particularly those in South America (e.g., Colombia [30/98, 30.6%], Bolivia [24/98, 24.5%], and Argentina [14/98, 41.3%]) exhibited a preference for phenytoin, as shown in Figure 2.

## 3 Discussion

In this multi‐center study, we found that 23% of patients with pediatric TBI had EPTS. Younger children and those with a GCS score of 8 or lower were more likely to have EPTS. The use of prophylactic ASM was associated with a lower prevalence of EPTS compared to those who did not.

The aim of brain protection strategies post‐TBI is to minimize secondary injury. Prophylactic ASM treatment has the potential to reduce the risk of EPTS occurrence [12]. During the 1990s, the findings of two seminal studies on epilepsy prevention after adult TBI were markedly different. A single‐center study of 404 adult TBI patients found that prophylactic ASM treatment reduced EPTS incidence compared to placebo [13]. However, another study with 179 TBI patients showed no difference in EPTS between groups treated with phenytoin versus placebo [14].

Due to the uncertainty regarding the effectiveness of prophylactic ASM therapy, there is a lack of consensus on its use. In a 2017 survey conducted by the UK and Ireland regarding the use of ASM, findings revealed that 53% of respondents did not consistently administer these agents. In contrast, 38% of physicians indicated they prescribed a 1‐week supply of prophylactic ASM. Additionally, 60% of participants expressed doubts about the efficacy of medications in preventing seizures [15]. Despite the increasing evidence base, the quality of the evidence remains inadequate due to limitations in research scale or scope. Meta‐analyses have examined EPTS in mild to moderate TBI cases [16, 17], and research has delved into risk factors for EPTS in children with moderate to severe TBI [7, 8, 17]. Our study, a comprehensive multicenter investigation of EPTS in children with mild to severe TBI in PICU, utilized the initial GCS score at hospital admission to reflect the children's baseline neurological status. The results indicated that 40.2% of children received prophylactic ASM. Although 117 children in our research initially presented GCS scores of 13–15, with 17 of them developing EPTS, representing 14.5%. We observed that children with a GCS score of 8 or lower are at increased risk of developing EPTS. In our survey, 62.1% of patients developed EPTS on the first day following TBI, with most experiencing seizures within the first 3 days. Prompt recognition and termination of the seizure cascade can effectively mitigate the sustained seizure activity [18, 19, 20]. The high incidence of seizures on the first day may partly result from suboptimal ASM levels at admission. Future studies should consider standardized ASM protocols to address this potential bias.

According to the Centers for Disease Control and Prevention (CDC) in the United States, the etiology of pediatric TBI differs by age, particularly at age 4 years old [21]. In our study, classification based on median age revealed that children aged ≤ 4 years were at an increased risk of developing EPTS. Prior studies have consistently shown that children aged 0–4 years are particularly vulnerable to TBI [22, 23]. At this age, children have delicate bodies and relatively larger heads, making them more susceptible to injuries from falls or impacts [23]. Their high levels of physical activity, combined with limited self‐protection awareness, further increase the risk of accidental injuries. Moreover, children aged 0–4 years are particularly vulnerable to abusive head trauma (AHT) due to their immature brain development and fragile nervous systems. Their complete dependence on adult caregivers heightens this risk, especially as abusive behaviors are often mistaken for accidental injuries, leading to an underestimation of AHT incidence and difficulties in early identification [21, 23, 24].

We found that phenytoin, levetiracetam, and phenobarbital were the most commonly used prophylactic ASM. Drug preferences varied significantly by country and region. South American countries favored phenytoin, whereas levetiracetam was preferred over phenytoin in Asia, particularly in China and Singapore. This trend mirrors that observed in Europe [25]. The incidence of EPTS was lower in Latin America than in Asia, potentially due to differences in the proportion of prophylactic ASM use and medication choices, such as the higher prevalence of phenobarbital use in some Asian countries. Future studies should conduct more detailed analyses to further explore these factors across different sites.

Phenytoin has traditionally been the mainstay in pediatric patients for preventing EPTS [13, 26]. However, its status as a first‐line ASM has diminished due to its adverse effects compared to levetiracetam [27, 28, 29]. A retrospective analysis also revealed that early use of levetiracetam was more beneficial in reducing the risk of EPTS in children compared to phenytoin [30, 31]. In our analysis of three ASMs, we evaluated potential confounding factors, including age, GCS score, and injury type. For children aged 4 years or younger, there appears to be an association between age and EPTS. However, the lack of statistical significance in the adjusted model suggests that age may not be a primary factor.

The differences in our study results compared to previous studies may be attributed to several factors. Variations in patient demographics, including sample size, age distribution, and injury mechanisms, as well as variations in dosing and timing of medication administration, can significantly influence treatment outcomes and drug efficacy [31]. Additionally, healthcare practitioners' familiarity with the necessary dosing and ability to manage these medications effectively can also impact clinical effectiveness. Interestingly, we found that a higher proportion of male patients received prophylactic treatment compared to female patients. This may be due to lower GCS scores in male children (7.9 vs. 8.1 for females) and prior research indicating that males have a higher risk of PTS, which may influence clinicians' prescribing practices [32]. Future studies should explore how different drug characteristics, such as dosing schedules and administration timing, interact with patient profiles to enhance clinical treatment strategies.

Our current study has several limitations. First, not all patients underwent continuous EEG monitoring, resulting in diagnoses primarily relying on observable clinical seizures. This methodology could have potentially underestimated the presence of subclinical seizures in the findings. Moreover, the lack of defined professional, centralized protocol for EEG data analysis may have introduced variability in interpretation across centers. Second, this study utilized a retrospective database design with non‐randomized treatment allocation. One limitation was the lack of detailed records regarding the prescribed ASM, including dosage, administration route, drug levels, and daily seizure frequency. For example, some patients may not have reached optimal drug levels on the first day of treatment but still experienced seizures. This highlights the potential challenges in interpreting the efficacy of prophylactic treatment in the absence of established therapeutic dosing or drug levels. Third, the study did not differentiate specific seizure types; given the varying ASM efficacy across seizure types, comparisons among the three drugs are limited. Fourth, regional heterogeneity in patient characteristics and ASM usage constitutes a limitation: PACCMAN (primarily Asia) featured higher proportions of patients, which may constrain extrapolation to other areas. Finally, since we performed an observational study, we were unable to establish causality. Future research should include more diverse cohorts, detailed ASM records (e.g., dosage, levels, and adverse events), standardized EEG monitoring with expert interpretation, and seizure type differentiation to better inform prophylactic ASM decisions and enhance generalizability.

## 4 Materials and Methods

### 4.1 Study Setting, Design, and Ethics

We performed a retrospective analysis of a multicenter observational TBI research database that had been previously established by two major PICU networks—the Pediatric Acute and Critical Care Medicine Asian Network (PACCMAN) and the Red Colaborativa Pediátrica de Latinoamérica (LARed Network), from January 2014 to December 2022 in tertiary care hospitals across 15 countries. In January 2021, LARed officially joined the research. The countries included those from Asia (Chinese Mainland and Hong Kong, Malaysia, Singapore, Pakistan, Japan, Saudi Arabia, and Thailand), Latin America (Mexico, Costa Rica, Colombia, Peru, Bolivia, Paraguay, and Argentina), and Europe (Spain). Sites were eligible to participate if they had a neurosurgical service on site.

### 4.2 Inclusion and Exclusion Criteria

Individuals < 18 years old who were admitted to the PICU with TBI were included in the study. EPTS are defined as seizures within 7 days of head trauma [33]. Patients with pre‐existing brain injuries, neurological diseases (regardless of concurrent use of ASM), late PTS (defined as seizure occurring 7 days or more after TBI), and those with incomplete data on important factors like the occurrence of seizure or prophylactic use of ASM were excluded.

### 4.3 Supplementary Definition

We obtained the initial Pediatric Glasgow Coma Scale (GCS) when the patients arrived at PICU. TBI was categorized into mild (GCS 13–15), moderate (GCS 9–12), and severe (GCS ≤ 8) based on the GCS at admission [34]. Prophylactic ASM is defined as those given before the first seizure. Patients who had seizures before ASM were assigned to the non‐prophylactic ASM EPTS group, while those with seizures after initiation were placed in the prophylactic ASM EPTS group. The selection of ASM was based on the discretion of the medical team and could vary according to the geographical location. Clinical and electrographic seizures (ESz) were recorded in the medical or nursing files and/or by electroencephalogram (EEG), with definitions based on the ACNS criteria [35, 36]. Clinical seizure is an epileptic event characterized by overt clinical symptoms, including motor manifestations, non‐motor symptoms, and autonomic changes. ESz is defined as either epileptiform discharges averaging > 2.5 Hz for ≥ 10 s (> 25 discharges in 10 s) or any pattern with definite evolution as defined above and lasting ≥ 10 s.

### 4.4 Variables and Data Management

Data were collected using a standardized electronic data from REDCap (Vanderbilt University). Data variables included patient demographics, such as sex and age, GCS score on PICU admission, occurrence of EPTS, the use of prophylactic ASM, presence of skull fracture, intracranial hemorrhage, penetrating TBI and multiple traumas, clinical treatment, including surgical intervention, targeted temperature management (with a threshold of 36°C), hypertonic therapy, and hyperventilation within 24 h. We presented age as a binary variable based on the median age of the study population dividing between those > 4 years old and those ≤ 4 years old, according to previous studies [37].

### 4.5 Statistical Analysis

Categorical variables are presented as frequencies and percentages, while continuous variables are expressed as either the mean with standard deviation (SD) or median with interquartile range (IQR), depending on the normality of distribution. Comparisons between the EPTS and non‐EPTS groups, as well as between the prophylactic ASM and non‐prophylactic ASM groups, were conducted using the chi‐square test. A multivariable logistic regression analysis was performed to evaluate the relationship between prophylactic ASM and EPTS, incorporating covariates chosen based on previous research [38, 39] and univariate logistic regression analysis (*p* < 0.2) from the initial assessment of potential predictors. In our multivariable regression analysis, we assessed multicollinearity using the variance inflation factor (VIF), ensuring all variables had VIF values below 10 to rule out significant multicollinearity. To assess the robustness of our findings, we conducted sensitivity analyses restricted to cases with EEG monitoring and stratified by region (PACCMAN and LARed). We presented both unadjusted and adjusted odds ratio (aOR) with their corresponding 95% confidence interval (95% CI). All statistical analyses were performed using SPSS software, version 22.0 (IBM, Armonk, NY, USA), with a *p*‐value of < 0.05 indicating statistical significance.

## 5 Conclusion

In conclusion, we found that use of prophylactic ASM was linked to a reduced odd of developing EPTS, while age ≤ 4 years and GCS ≤ 8 were associated with an elevated incidence of EPTS in pediatric TBI. This multicenter study also reports region‐specific differences in the use and choice of prophylactic ASM. Future randomized controlled trials are required to test the efficacy of prophylactic ASM in the setting of pediatric TBI.

## Author Contributions

S.Q.Y and J.J. contributed to the concept and design; J.J., J.D.R.G., S.‐L.C., Q.W., C.S.G., J.P.W.N., T.E.T.M., F.I.P.C., L.C.N.C., Q.A., J.S.M.O., I.J.A., Y.M.K., J.A.D.‐R., H.K., S.B.R.D., N.E.G.A., N.Z.W., A.Y.‐C., R.E.L.P., G.A., P.C., R.E.d.l.T.G., C.L., R.S., N.A., D.M.T., M.F.F., P.‐C.L., F.J.P.‐O., H.D., Y.K.C., M.M., J.C.J.‐B., S.G.‐D., J.H.L., and S.Q.Y. contributed to acquisition of data; J.J., S.‐L.C., and Q.W. contributed to the analysis and interpretation of data; S.‐L.C., J.H.L., Y.M.K., Q.A., P.C., J.A.D.‐R., and S.G.‐D. contributed to drafting the article or revising it critically for important intellectual content. All authors have read and approved the final manuscript.

## Funding

This study was funded by a SingHealth Foundation research grant, Singapore (SHF/FG670P/2017) and the National Medical Research Council (NMRC), Singapore (MOH‐CSSSP19may‐0020). The grants were awarded to Dr. Shu‐Ling Chong. The funding organization has no role in the design of the study, collection, analysis, interpretation of data, or in writing of the manuscript. This study was also funded by Establishing a Monitoring System for Cerebral Blood Flow and Cerebrovascular Auto‐regulation in Children with Severe TBI (YN2020407). The grants were awarded to pediatric intensive care unit of Beijing children's Hospital. The funding organization has no role in the design of the study, collection, analysis, interpretation of data, or in writing of the manuscript.

## Ethics Statement

Ethics approval for the multicenter observational study was obtained from the SingHealth Centralized Institutional Review Board (CIRB) in Singapore (CIRB 2018/2457, June 1, 2018, Title: Does 3% hypertonic saline decrease mortality and improve long‐term neurological outcomes among children with traumatic brain injury? and CIRB 2018/2076, Feb 8, 2018, Title: Variation in intensive care practices for moderate to severe traumatic brain injury: A multi‐national initiative). Additional approval was secured from the ethics boards of each participating center, along with a waiver for informed consent. Procedures were in accordance with the ethical standards of the responsible committee on human experimentation (institutional) and with the Helsinki Declaration of 1975.

## Conflicts of Interest

The authors declare no conflicts of interest.

## Supporting information




**Table S1**: Sensitivity analysis of multivariable logistic regression evaluating risk factors for EPTS in the PACCMAN region
**Table S2**: Sensitivity analysis of multivariable logistic regression evaluating risk factors for EPTS in the LARed region
**Table S3**: Sensitivity analysis of multivariable logistic regression evaluating risk factors for EPTS in the EEG‐monitored patients
**Table S4**: Multivariable logistic regression analysis of three ASMs

## Data Availability

The datasets generated during the current study are available from the corresponding author upon reasonable request.
