# Supplementary material for: Association Between Prophylactic Anti‐Seizure Medication and Early Post‐Traumatic Seizures: An International Observational Multicenter Study
Source: MedComm (2020). 2025 Dec 10;6(12):e70524. doi: 10.1002/mco2.70524 (PMC12696337; doi:10.1002/mco2.70524)
Supplement: Supplementary file 1 — Table S1: Sensitivity analysis of multivariable logistic regression evaluating risk factors for EPTS in the PACCMAN region Table S2: Sensitivity analysis of multivariable logistic regression evaluating risk factors for EPTS in the LARed region Table S3: Sensitivity analysis of multivariable logistic regression evaluating risk factors for EPTS in the EEG‐monitored patients Table S4: Multivariable logistic regression analysis of three ASMs [file MCO2-6-e70524-s001.docx]

**Association between prophylactic Anti-seizure medication and early post-traumatic seizures: An international observational multicenter study**

***Short title:*** Prophylactic Antiepileptics and EPTS in children

**Author names:** Jian Ji^1^,Juan D. Roa G^2^, Shu-Ling Chong^3^, Quan Wang^4^, Chin Seng Gan^5^, Jane P.W Ng^6^, Thelma Elvira Teran Miranda^7^, Freddy Israel Pantoja Chamorro^8^, Lawrence Chi Ngong Chan^9^, Qalab Abbas^10^,Jacqueline S.M.Ong^11^, Ivan J.Ardila^12^, Yasser M. Kazzaz^13^, Jesús A.Domínguez-Rojas^14^, Hiroshi Kurosawa^15^,Susana Beatriz Reyes Domínguez^16^, Natalia Elizabeth Gómez Arriola^17^, Natalia Zita Watzlawik^18^, Adriana Yock-Corrales^19^, Rubén Eduardo Lasso Palomino^20^, Gabriela Aparicio^21^, Paula Caporal^22^, Rosa Elena de la Torre Gómez^23^, Chunfeng Liu^24^, Rujipat Samransamruajkit^25^, Nattachai Anantasit^26^, Deborah M.Turina^27^, Marisol Fonseca Flores^28^, Pei-Chuen Lee^29^, Francisco J. Pilar-Orive^30^, Hongxing Dang^31^,Yek Kee Chor^32^, Meixiu Ming^33^, Juan C.Jaramillo-Bustamante^34^, Sebastián González-Dambrauskas^35^, Jan Hau Lee^36^*, Suyun Qian^1^*

**Author affiliations:**

1. Pediatric Intensive Care Unit, Beijing Children’s Hospital, Capital Medical University, National Center for Children`s Health, Beijing, China
2. Pediatric Collaborative Latin American Network (LARed Network); Pediatric Intensive Care Unit, Los Cobos Medical Center, Universidad del Bosque, Bogotá, Colombia
3. Department of Emergency Medicine, KK Women’s and Children’s Hospital, Singapore; SingHealth Duke-NUS Global Health Institute, Duke-NUS Medical School, Singapore
4. Pediatric Intensive Care Unit, Beijing Children’s Hospital, Capital Medical University, National Center for Children`s Health, Beijing, China
5. Department of Pediatrics, University Malaya Medical Centre, Kuala Lumpur, Malaysia
6. KK Research Centre, KK Women`s and Children`s Hospital, Singapore
7. Department of Pediatrics, Hospital del Niño Manuel Ascencio Villarroel, Cochabamba, Bolivia
8. Intensive Care Unit, Hospital Infantil Los Angeles, Pasto, Colombia
9. Department of Pediatrics, Prince of Wales Hospital, The Chinese University of Hong Kong, Hong Kong S.A.R, China
10. Department of Pediatrics and Child Health, Aga Khan University Hospital, Karachi, Pakistan
11. Khoo Teck Puat National University Children’s Medical Institute, National University Hospital, Singapore; Department of Pediatrics, Yong Loo Lin School of Medicine, National University of Singapore, Singapore
12. Pediatric Critical Care, Clinica UROS, Neiva, Huila, Colombia
13. Department of Pediatrics, King Abdullah International Medical Research Center, Riyadh, Saudi Arabia
14. Department of Pediatrics and Pediatric Critical Care Medicine, Hospital Nacional Hipolito Unanue, El Agustino, Peru; Pediatric Chapter of the Peruvian Society of Intensive Care Medicine, Lima, Peru
15. Department of Pediatric Critical Care Medicine, Hyogo Prefectural Kobe Children’s Hospital, Kobe, Japan
16. Pediatric intensive Care Unit, Pediatric Department, Virgen de la Arrixaca Hospital, Murcia, Spain
17. Emergency Department, Hospital del Trauma, Asunción, Paraguay
18. Department of Pediatric Intensive Care Unit, Hospital de Pediatric Garrahan, Ciudad Autónoma de Buenos Aires, Argentina
19. Emergency Department, National Children's Hospital "Dr. Carlos Saenz Herrera," CCSS, San José, Costa Rica
20. Pediatric Intensive Care Unit, Fundación Valle del Lili, Cali, Valle del Cauca, Colombia
21. Pediatric Critical Care Unit, Hospital de Ninos de La Plata Sor María Ludovica, Buenos Aires, Argentina
22. Pediatric Intensive Care Unit, Children's Hospital "Sor Maria Ludovica," Buenos Aires, Argentina; Pediatric Collaborative Latin American Network (LARed Network)
23. Hospital Civil de Guadalajara, Guadalajara, Mexico
24. Pediatric Department, Shengjing hospital, China Medical University, Shenyang, Liaoning, China
25. Department of Pediatrics, Pediatric Critical Care Division, King Chulalongkorn Memorial Hospital, Chulalongkorn University, Bangkok, Thailand
26. Pediatric Critical Care Division, Department of Pediatrics, Ramathibodi Hospital, Bangkok, Thailand
27. Pediatric intensive Care Unit, Ricardo Guiterrez Children's Hospital, Buenos Aires, Argentina
28. Pediatric Critical Care, Mexican Institute of Social Security, México City, Mexico
29. Pediatrics Department, UKM Specialist Children's Hospital, Kuala Lumpur, Wilayah Persekutuan, Malaysia
30. Department PICU, Hospital Universitario de Cruces, Spain
31. Department of Pediatric Intensive Care Unit, Children’s Hospital of Chongqing Medical University, Chongqing, China
32. Department of Pediatrics, Sarawak General Hospital, Sarawak, Malaysia
33. Department of Pediatric Intensive Care Unit, Children’s Hospital of Fudan University, Shanghai, China
34. MD, Pediatric Intensive Care Unit, General Hospital of Medellín “Luz Castro de Gutiérrez,” Medellín, Colombia
35. Pediatric Collaborative Latin American Network (LARed Network); Department of Pediatrics and Pediatric Intensive Care Unit, Hospital Pereira Rossell, School of Medicine, University of the Republic, Montevideo, Uruguay
36. Children’s Intensive Care Unit, KK Women’s and Children’s Hospital, Singapore

***Correspondence:** Jan Hau Lee, MBBS, MRCPCH, MCI. Children’s Intensive Care Unit, KK Women’s and Children’s Hospital, 100 Bukit Timah Road, 229899 Singapore. Email: [gmsljh@nus.edu.sg](mailto:gmsljh@nus.edu.sg);

Suyun Qian, MD, Pediatric Intensive Care Unit, Beijing Children’s Hospital, Capital Medical University, National Center for Children’s Health, No.56 Nan Li-Shi Road, Beijing, 100045, China. Email: [syqian2020@163.com](mailto:syqian2020@163.com).

**Table S1. Sensitivity analysis of multivariable logistic regression evaluating risk factors for EPTS in the PACCMAN region**

| **Characteristics** | **Unadjusted OR (95% CI)** | **Unadjusted *p* value** | **Adjusted OR (95% CI)** | **Adjusted *p* value** |
| --- | --- | --- | --- | --- |
| ≤4 years old | 3.32 (2.13, 5.28) | <0.001 | 2.96 (1.59, 5.65) | <0.001 |
| GCS ≤8 | 1.36 (0.91, 2.04) | 0.138 | 1.53 (1.31, 1.75) | <0.001 |
| Skull fracture | 0.59 (0.33, 1.00) | 0.057 | 0.96 (0.43, 2.04) | 0.308 |
| Intracranial hemorrhage | 1.28 (0.95, 1.68) | 0.062 | 1.37 (0.89, 2.13) | 0.143 |
| Multiple trauma | 0.70 (0.47, 1.04) | 0.081 | 1.02 (0.55, 1.89) | 0.961 |
| Hypertonic therapy | 0.78 (0.50, 1.22) | 0.266 | 0.53 (0.27, 1.04) | 0.066 |
| Temperature control | 1.08 (1.00, 1.18) | 0.061 | 1.06 (0.94, 1.20) | 0.940 |
| Prophylactic ASM | 0.59 (0.47, 0.71) | <0.001 | 0.46 (0.39, 0.53) | <0.001 |
| Abbreviation: GSC, Glasgow Coma Scale; ASM, anti-seizure medication. | | | | |

**Table S2. Sensitivity analysis of multivariable logistic regression evaluating risk factors for EPTS in the LARed region**

| **Characteristics** | **Unadjusted OR (95% CI)** | **Unadjusted *p* value** | **Adjusted OR (95% CI)** | **Adjusted *p* value** |
| --- | --- | --- | --- | --- |
| ≤4 years old | 1.07 (1.02, 1.12) | <0.001 | 2.23 (1.10, 3.65) | <0.001 |
| GCS ≤8 | 1.82 (0.86, 3.90) | 0.117 | 2.33 (1.89, 2.75) | <0.001 |
| Skull fracture | 0.79 (0.33, 1.25) | 0.153 | 0.71 (0.43, 3.03) | 0.431 |
| Intracranial hemorrhage | 1.29 (0.52, 3.67) | 0.603 | 5.89 (0.92, 45.73) | 0.072 |
| Multiple trauma | 0.84 (0.36, 1.85) | 0.676 | 0.32 (0.05, 1.54) | 0.172 |
| Hypertonic therapy | 0.68 (0.32, 1.47) | 0.321 | 1.34 (0.32, 6.28) | 0.696 |
| Temperature control | 0.71 (0.35, 1.01) | 0.148 | 0.89 (0.38, 1.55) | 0.720 |
| Prophylactic ASM | 0.13 (0.07, 0.19) | <0.001 | 0.26 (0.19, 0.35) | <0.001 |
| Abbreviation: GSC, Glasgow Coma Scale; ASM, anti-seizure medication. | | | | |

**Table S3. Sensitivity analysis of multivariable logistic regression evaluating risk factors for EPTS in the EEG-monitored patients**

| **Characteristics** | **Unadjusted OR (95% CI)** | **Unadjusted *p* value** | **Adjusted OR (95% CI)** | **Adjusted *p* value** |
| --- | --- | --- | --- | --- |
| ≤4 years old | 2.18 (1.21, 4.00) | 0.011 | 2.24 (1.94, 2.48) | 0.021 |
| GCS ≤8 | 1.69 (0.88, 2.50) | 0.206 | 1.80 (1.18, 2.74) | 0.010 |
| Skull fracture | 1.14 (0.57, 2.27) | 0.701 | 2.95 (0.97, 9.48) | 0.060 |
| Intracranial hemorrhage | 1.25 (0.70, 2.27) | 0.457 | 2.50 (0.96, 6.93) | 0.067 |
| Multiple trauma | 0.73 (0.41, 1.30) | 0.285 | 1.36 (0.58, 3.26) | 0.479 |
| Hypertonic therapy | 0.58 (0.27, 1.25) | 0.159 | 0.52 (0.16, 1.58) | 0.253 |
| Temperature control | 0.97 (0.86, 1.10) | 0.635 | 0.98 (0.63, 1.33) | 0.940 |
| Prophylactic ASM | 0.37 (0.16, 0.58) | <0.001 | 0.23 (0.16, 0.30) | <0.001 |
| Abbreviation: GSC, Glasgow Coma Scale; ASM, anti-seizure medication. | | | | |

**TableS4. Multivariable logistic regression analysis of three ASMs**

| **Characteristics** | **Unadjusted odds ratio (95% CI)** | **Unadjusted *p* value** | **Adjusted Odds ratio (95% CI)** | **Adjusted *p* value** |
| --- | --- | --- | --- | --- |
| ≤4 years old | 2.43 (1.00, 5.88) | 0.05 | 2.36 (0.89, 6.20) | 0.08 |
| GCS ≤8 | 0.73 (0.31, 1.72) | 0.48 | 0.56 (0.21, 1.48) | 0.24 |
| Sex | 1.56 (0.67, 3.60) | 0.30 | 1.41 (0.56, 3.58) | 0.47 |
| Multiple trauma | 1.23 (0.53, 2.86) | 0.63 | 1.45 (0.56, 3.71) | 0.44 |
| Skull fracture | 0.86 (0.31, 2.41) | 0.78 | 0.65 (0.20, 2.17) | 0.49 |
| Intracranial hemorrhage | 2.05 (0.68, 6.19) | 0.21 | 2.56 (0.76, 8.61) | 0.13 |
| Hypertonic therapy | 1.40 (0.39, 5.04) | 0.61 | 1.78 (0.37, 8.61) | 0.47 |
| Prophylactic ASM |  |  |  |  |
| Phenytoin | Ref. |  | Ref. |  |
| levetiracetam | 0.12 (0.04, 0.38) | ＜0.001 | 0.11 (0.03, 0.3) | ＜0.001 |
| Phenobarbital | 0.26 (0.09, 0.70) | 0.01 | 0.23 (0.08, 0.67) | 0.01 |
| Abbreviation: GSC, Glasgow Coma Scale; ASM, anti-seizure medication. | | | | |
